# Membrane-Mediated Interaction between Strongly Anisotropic Protein Scaffolds

**DOI:** 10.1371/journal.pcbi.1004054

**Published:** 2015-02-24

**Authors:** Yonatan Schweitzer, Michael M. Kozlov

**Affiliations:** Department of Physiology and Pharmacology, Sackler Faculty of Medicine, Tel Aviv University, Tel Aviv, Israel; Pierre and Marie Curie University (UPMC), UNITED KINGDOM

## Abstract

Specialized proteins serve as scaffolds sculpting strongly curved membranes of intracellular organelles. Effective membrane shaping requires segregation of these proteins into domains and is, therefore, critically dependent on the protein-protein interaction. Interactions mediated by membrane elastic deformations have been extensively analyzed within approximations of large inter-protein distances, small extents of the protein-mediated membrane bending and small deviations of the protein shapes from isotropic spherical segments. At the same time, important classes of the realistic membrane-shaping proteins have strongly elongated shapes with large and highly anisotropic curvature. Here we investigated, computationally, the membrane mediated interaction between proteins or protein oligomers representing membrane scaffolds with strongly anisotropic curvature, and addressed, quantitatively, a specific case of the scaffold geometrical parameters characterizing BAR domains, which are crucial for membrane shaping in endocytosis. In addition to the previously analyzed contributions to the interaction, we considered a repulsive force stemming from the entropy of the scaffold orientation. We computed this interaction to be of the same order of magnitude as the well-known attractive force related to the entropy of membrane undulations. We demonstrated the scaffold shape anisotropy to cause a mutual aligning of the scaffolds and to generate a strong attractive interaction bringing the scaffolds close to each other to equilibrium distances much smaller than the scaffold size. We computed the energy of interaction between scaffolds of a realistic geometry to constitute tens of k_B_T, which guarantees a robust segregation of the scaffolds into domains.

## Author Summary

A striking feature of most of the crucial intra-cellular organelles, such as Endoplasmic Reticulum (ER), Golgi Complex and mitochondria is a peculiar and strongly curved shape of their membranes. Membranes resist elastically to bending and remodeling. Hence, special proteins must generate forces needed to create and maintain the intracellular membrane curvature and architecture. An effective mechanism by which proteins bend membranes is the scaffolding mechanism based on attachment to the outer membrane surface of hydrophilic protein domains referred to as a protein scaffolds. The scaffolds are intrinsically bent and mold, therefore, the membranes into curved shapes. This action of the scaffolds requires their concentration on the membrane surface due to inter-scaffold attractive forces originating from membrane deformations. Here we analyze quantitatively the membrane-mediated interaction between two strongly anisotropic and intrinsically curved membrane scaffolds, which mimic realistic proteins. We demonstrate that the scaffold shape anisotropy causes a mutual aligning of the scaffolds and generates a strong attractive interaction bringing the scaffolds close to each other to equilibrium distances much smaller than the scaffold size. We compute the energy of interaction between scaffolds of a realistic geometry to constitute tens of *k*_B_ T, which guarantees a robust segregation of the scaffolds into domains.

## Introduction

Interactions between membrane shaping proteins play a critical role in sculpting intracellular organelles [1].

Membranes of such cellular compartments in as endoplasmic reticulum (ER), Golgi Complex, mitochondria and transport intermediates possess peculiar shapes containing strongly curved regions with radii of few tens of nanometers [2]. Also upon pathologic conditions of, e.g., viral infection intracellular membrane can develop strongly curved abnormal structures similar to lyotropic cubic phases [3,4]. While membrane curvature can be a consequence of a difference in lipid composition of the two membrane monolayers [5,6] or membrane pulling by molecular motors [7,8], it is commonly agreed that in most cases large curvatures characterizing the intracellular membranes are generated and stabilized by specialized peripheral membrane proteins (see for recent reviews [9–11]). It has been further suggested that, in addition to the curvature *per se*, proteins can generate also anisotropy of membrane shapes and elastic properties [12,13].

The two mechanisms suggested for generation of large membrane curvatures by individual membrane proteins or protein oligomers are the hydrophobic insertion (wedging) and scaffolding mechanisms (see for recent review [11]). The essence of the hydrophobic insertion mechanism is a shallow embedding into the external membrane monolayer of small hydrophobic or amphipathic domains of such proteins as epsins [14], N-BAR domain proteins [15,16] and ALPS motifs [17]. One such insertion serves as an effective wedge splaying the monolayer polar heads, which generates a local perturbation of the membrane structure [11]. Perturbations by numerous insertions concentrated within one spot in the membrane plane sum up into an overall membrane curvature [11,16].

The scaffolding mechanism is based on attachment to the outer membrane surface of a hydrophilic protein domain or a domain oligomer, referred to as a protein scaffold, which molds the membrane, locally, into a curved shape [7,8,18]. This mechanism requires the membrane binding face of the scaffold to be intrinsically curved, the protein-membrane interaction of, usually, electrostatic origin, to be sufficiently strong, and the scaffold to be more rigid that the lipid membrane matrix. Similarly to a hydrophobic insertion, a protein scaffold provides a local membrane deformation so that generation of membrane curvature along an extended membrane area requires an elevated surface density of such scaffolds.

The protein segregation on the membrane surface into regions of high protein densities must be driven by attractive forces between the individual protein or their oligomers. Whereas direct electrostatic, Van-der Waals, and liquid crystal-like interactions may contribute to such forces [1], an essential protein-protein interaction can be mediated by the membrane itself (see for recent review [19]). Theoretical and numerical analyses of such membrane-mediated interactions are necessary for determination of conditions where these interactions become relevant for the in-plane protein assembly, i.e., are attractive and strong enough to overcome the entropic spreading of the proteins over the membrane surface.

The models suggested in the literature for analysis of the membrane-mediated interactions between proteins are of a phenomenological or microscopic character [20–23]. The phenomenological model considers a protein either as a point-like perturbation of the membrane elastic properties [23] or as a membrane patch whose bending modulus, *κ*_*p*_ and the modulus of Gaussian curvature, ${\bar{\kappa }}_{p}$, [24], only slightly deviate from those of the surrounding lipid bilayer, *κ* and $\bar{\kappa }$, so that $\frac{\left|\kappa_{p}-\kappa \right|}{\kappa }\ll 1$, $\frac{\left|{\bar{\kappa }}_{p}-\bar{\kappa }\right|}{\left|\bar{\kappa }\right|}\ll 1$. The energies of the membrane conformations, which determine the overall membrane free energy and the related protein-protein interaction, are computed in this case by integration of Helfrich Hamiltonian of membrane bending [24] over the total membrane area including the protein patches, and the results are presented as series in $\frac{\left|\kappa_{p}-\kappa \right|}{\kappa }$ and $\frac{\left|{\bar{\kappa }}_{p}-\bar{\kappa }\right|}{\left|\bar{\kappa }\right|}$ [21,22]. The microscopic models assume the proteins to be infinitely rigid, as compared to the membrane either in terms of their bending rigidity, $\frac{\kappa_{p}}{\kappa }\gg 1$, or including also the rigidity with respect to Gaussian curvature $\frac{\left|{\bar{\kappa }}_{p}\right|}{\left|\bar{\kappa }\right|}\gg 1$ [25]. In this case, the protein patches do not accumulate any bending energy but rather impose along their perimeters certain boundary conditions on the membrane shape [21,22]. Two types of such boundary conditions have been introduced. The first type sets a particular contact angle, *ϕ*, between the membrane and the protein patch [21,22,26–29], which is supposed to be determined by an effective shape of the protein-membrane interface. The contact angle has been assumed to have either a fixed [21,26–29] or an energetically preferred [22] value at every point of the protein-membrane boundary. The boundary condition of the second type assumes that the protein generates, locally, a certain mean curvature of the membrane surface [19,30](see for review [31]). Fulfillment of the latter condition would require the protein to produce a constant bending moment acting on the membrane along the protein circumference. Whereas existence of a molecular mechanism for such bending moment generation seems uncertain, the qualitative features of the membrane mediated protein-protein interactions appear to be insensitive to the particular type of the boundary conditions [30,31].

The model analysis revealed two different physical origins for the membrane-mediated interactions between proteins. The first is of entropic nature and is related to modification by the proteins of the thermal membrane undulations [21,22,31–35]. The second, referred to as the elastic interaction, is a consequence of the membrane bending deformation generated by the proteins [19–22,26–31,34,36–40]. In both cases the interaction character depends on the shape of the effective membrane inclusions representing the protein. For axially symmetric inclusions having shapes of spherical segments or truncated symmetric cones, the entropic interaction is purely attractive, while the elastic interaction, in the case of vanishing lateral tension, is pure repulsive, both decaying as 1 / *d* ^4^ for the inter-inclusion distances, *d*, greatly exceeding the inclusion size, *a* (see for review [38]).

Any kind of anisotropy in the inclusion shape changes qualitatively the character of the inter-inclusion interaction making it dependent on the inclusion orientation in the membrane plane. The considered types of such anisotropy are an elliptical rather than circular base shape of an inverted cone [22,28] with possible variations of the contact angle and of the level of the contact line with respect to the cone base [27], and a saddle-like rather spherical local curvature imprint generated by the inclusion [19,30]. Specifically, for certain inclusion orientations, the elastic interaction has been predicted to acquire an attractive component, which decay with the distance as 1 / *d* ^2^ for d ⪢ a [19,22,27,28,30,33].

While the previous extensive analysis revealed the major qualitative features of the membrane-mediated interactions between membrane inclusions, it was mainly performed (but [37]) for large inter-inclusion distances, d ⪢ a, low degrees of the inclusion anisotropy and in the approximation of small local perturbations by the inclusions of the initially flat membrane shape. In reality, these conditions are often violated. The crescent-like shapes of such typical curvature-generating protein scaffolds as BAR-domain dimers are strongly elongated having aspect ratio around five [15] and so strongly curved [7,41–44] that the contact angles at the effective membrane-protein boundary are large, hence, generating strong local perturbations of the membrane shape. Strongly elongated as well as strongly and anisotropically curved shapes characterize also oligomers of dynamin (see for review [45–48]) and EHD2 [49]. The inter-protein distances are hardly accessible for direct measurement within cells. However in *in vitro* systems [42] and, according to estimations, within strongly curved membrane domains such as ER tubules [9,50,51], these distances are comparable to the protein size, *d*~*a*. Thus, understanding of the biologically relevant protein-protein interactions requires expansion by numerical methods of the previously obtained results to the experimentally relevant parameter ranges.

The goal of the present study is to analyze quantitatively the membrane-mediated interaction between two strongly anisotropic and intrinsically curved membrane scaffolds, which mimic realistic proteins and protein oligomers shaping membrane by the scaffolding mechanism.

Following the information on the protein structures provided by crystallographic studies [52], we model a protein scaffold as a surface patch with infinite rigidity and an anisotropic, strongly and inhomogeneously curved shape. We first compute the elastic interaction between scaffolds whose footprint on the membrane plane is circular but the two principal curvatures are different. We calculate the orientational component of this interaction driving the scaffold mutual ordering in the membrane plane, and the repulsive-attractive component determining the preferred inter-scaffold distance. We demonstrate that the attractive interaction dominates at large distance between the scaffolds, while a repulsive interaction takes over at small distances. As a result, there is always an energy well corresponding to an equilibrium inter-scaffold distance, which is determined by the specific type and extent of the scaffold shape anisotropy. In a particular case of scaffolds with one vanishing principal curvature, the elastic interaction remains attractive up to vanishing distances. In addition to the purely elastic forces, we estimate, computationally, a previously unaddressed contribution of the entropy of fluctuations of the scaffold mutual orientation. We obtain that this contribution is always repulsive and is of the same order of magnitude as the entropic interaction related to the membrane undulations. Finally, we perform computations for strongly elongated and strongly and anisotropically curved protein scaffolds whose geometry is characterized by the specific parameters assessed experimentally for BAR proteins [15,41]. We find that these proteins prefer a parallel mutual orientation, and that the energy “well” corresponding to an inter-scaffold equilibrium distance much smaller than the protein width of several nanometers reaches few tens of k_B_T (where k_B_T = 410^–21^ is the product of Boltzmann constant and the absolute temperature). This means that the elastic interaction is sufficiently strong to drive the protein segregation and mutual ordering.

## Model

### System description

We consider two infinitely rigid protein scaffolds having an anisotropic shape and embedded into a flexible lipid membrane. Our goal is to compute the energy and the related force of interaction between the scaffolds determined by the membrane elasticity and the free energy contribution from the entropy of fluctuations of the scaffold mutual orientation.

**Fig 1:**
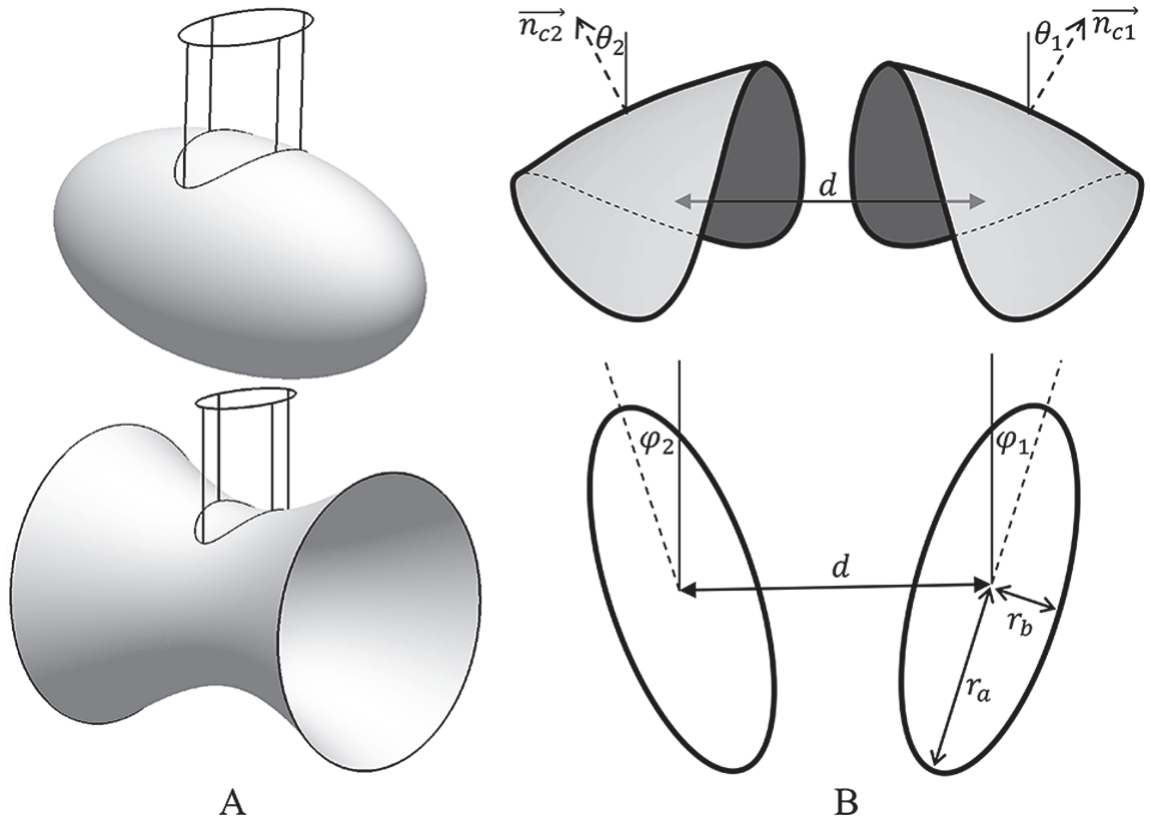
Schematic illustration of the scaffolds modeling and parameters in use. **(A**). The surfaces used for the scaffold shape derivation. Top—ellipsoid (*ρ* *_b_* > 0), bottom—hyperboloid (*ρ* *_b_* < 0). An intersection with an elliptical cylinder gives rise to a scaffold shape. (B). Scaffold shape and parameters: top—side view; bottom—projection of the initial membrane plane.

We model a scaffold as a segment of ellipsoid (or hyperboloid) (Fig. 1 A) with a given area *A*, different principal curvatures, *c*_*a*_ and *c*_*b*_, in the central point and an elliptical shape of projection on the initial membrane plane characterized by semi-axes *r*_*a*_ and *r*_*b*_ (Fig. 1B). Specifically, the scaffold shape is obtained by cutting a surface determined in Cartesian coordinates by

$${\left(\frac{x}{\rho_{a}}\right)}^{2}+sign\left(\rho_{b}\right){\left(\frac{y}{\rho_{b}}\right)}^{2}+{\left(\frac{z}{\rho_{a}}\right)}^{2}=1,$$ (1)

by a cylindrical surface whose axis of symmetry coincides with the axis *z* and the cross-section in the *x-y* plane is an ellipse determined by

$${\left(\frac{x}{r_{a}}\right)}^{2}+{\left(\frac{y}{r_{b}}\right)}^{2}=1.$$ (2)

The parameters *ρ*_*a*_, *ρ*_*b*_, *r*_*a*_ and *r*_*b*_ determine the scaffold area *A* and the principle curvatures *c*_*a*_ = 1/ *ρ*_*a*_, *c*_*b*_ = 1/ *ρ*_*b*_ in the central point of the scaffold. Positive values of *ρ*_*a*_ > and *ρ*_*b*_ > 0 correspond to ellipsoidal shapes of the scaffolds with two positive principal curvatures, whereas a positive *ρ*_*a*_ > 0 but negative *ρ*_*b*_ < 0 describe a hyperboloidal saddle-like shapes, whose principal curvatures have opposite signs (Fig. 1A).

To simulate a cell outer membrane, we model the initial membrane shape preceding its perturbation by the scaffolds as a closed sphere with radius *R*. This membrane radius is taken to exceed by four orders of magnitude the scaffold dimension, $a=\sqrt{A}$, and the inter-scaffold distances, *d*, meaning that, locally, in the vicinity of the scaffolds, the initial membrane shape is, practically, flat. In the course of deformations, the membrane is assumed to remain closed and keep its area, 4π*R* ^*2*^, but to match the enclosed volume to the energy minimization requirements.

As a boundary condition for the membrane shape imposed by a scaffold we require continuity between the membrane and scaffold surfaces, which means that at every point along the scaffold perimeter the normal vector of the membrane surface, ${\vec{n}}_{e}$, coincides with that of the scaffold surface (Fig. 1B). This boundary condition can be also expressed through the angle, *ϕ*, between the normal vector, ${\vec{n}}_{e}$, and that at the scaffold center, $\;{\vec{n}}_{c}$, which will be referred to as the contact angle (Fig. 1B). The contact angle changes along the scaffold perimeter in the range between *ϕ*_*a*_ = arcsin(*r*_*a*_ / *ρ*_*a*_) and *ϕ*_*b*_ = arcsin(*r*_*b*_ / *ρ*_*b*_).

Note that the curvature continuity is not required since the border of the scaffold can apply bending moments to the membrane.

Because of the infinite rigidity, the scaffolds do not accumulate any elastic energy. The elastic energy of the membrane per unit area of the membrane surface is described by Helfrich model [24]

$$f=\frac{1}{2}\kappa \;J^{2},$$ (3)

where *κ* is the membrane bending modulus [24], whose value will be taken as *k* = 20 *k*_*B*_ *T*, and *J* is the total curvature of the membrane surface (which equals twice the mean curvature) [53]. The (Eq. 3) implies that the membrane spontaneous curvature vanishes; the energy of Gaussian curvature is constant according to Gauss-Bonnet theorem since the direction of the surface normal is fixed at the scaffold boundaries and the membrane surface remains closed; and there is no lateral tension in the membrane. Below we show that the latter assumption is not crucial and the following computation results account also for the cases of non-vanishing lateral tension, *γ*, provided that the related characteristic length of propagation of membrane deformations, $\sqrt{\kappa /\gamma }$, greatly exceeds the scaffold size and the inter-scaffold distance.

The total elastic energy is obtained by integration of Eq. 3 over the area *A* of the closed membrane exept for the two segments occupied by the scaffolds,

$$F_{m}=\int^{\text{​}}f\;dA.$$ (4)

We determine the relative positions and orientations of the two scaffolds on the membrane by the distance *d* between the scaffold centers, the angles *φ*_*1*_ and *φ*_*2*_ between the scaffold long axes and the line connecting the scaffold centers, and the angles *θ*_*1*_ and *θ*_*2*_ of tilting of the normal vectors at the scaffold centers, ${\vec{n}}_{c1}$ and ${\vec{n}}_{c2}$, with respect to the normal vector of the initial membrane plane (Fig. 1B). For simplicity, we consider only symmetric orientations of the scaffolds *φ*_*1 =*_ *φ*_*2 ≡*_ *φ* and *θ*_*1 =*_ *θ*_*2 ≡*_ *θ* (Fig. 1). Below we support this assumption by computing the membrane energy for several characteristic asymmetric orientations. Because of the boundary conditions at the scaffold perimeters, at each specific distance, *d*, and orientation angles, *φ* and *θ*, the membrane undergoes bending deformation, and acquires an elastic energy, *F*_*m*_(*φ*, *θ*, *d*).

### Energy of the scaffold interaction

We define as the *elastic energy* of the membrane-mediated scaffold interaction, *F*_*el*_(*d*), the membrane elastic energy, *F*_*m*_(*φ*, *θ*, *d*), which is minimized for every inter-scaffold distance, *d*, with respect to the angles *φ* and *θ*. The angle values *φ* ^*^(*d*) and *θ* ^*^(*d*) corresponding to the minimal energy characterize the scaffold mutual alignment.

Further, taking the angles *φ* and *θ* as variables determining the thermodynamic states of the system for any inter-scaffold distance, *d*, we define the *free energy* of the membrane-mediated interaction between the scaffolds as

$$F\left(d\right)=-k_{B}T\log\left[\int^{\text{​}}\exp\left(-\frac{F_{m}\left(\varphi ,\theta ,d\right)}{k_{B}T}\right)d\theta d\varphi \right]$$ (5)

where the integration has to be performed over the whole range of the angle variations 0 < *φ <* 90°, 0 < *θ <* 180°.

Finally, taking the elastic energy, *F*_*el*_(*d*), as playing the role of the internal energy of the system, we define the *entropic energy* of the membrane-mediated scaffold interaction, *F*_*ent*_(*d*), as

$$F_{ent}\left(d\right)=F\left(d\right)-F_{el}(d).$$ (6)

Note that the entropic energy we consider (Eq. 6) does not include the contribution of the membrane undulations analyzed in the previous works [20,21,31,33,38] and is related solely to the fluctuations of the scaffold orientation. Furthermore, as discussed below, computations within the limitation of the symmetric mutual orientations of the scaffolds give a low limit of the related entropic interaction.

The interaction forces acting between the scaffolds and corresponding to the free, elastic and entropic energies are the derivatives of *F*(*d*), *F*_*el*_(*d*) and *F*_*ent*_(*d*) with respect to the inter-scaffold distance, *d*.

**Way of computation**. For computations we use K. Brakke’s Surface Evolver [54]. For every given set of the inter-scaffold distance, *d*, and the orientation angles, *φ* and *θ*, we obtain the membrane bending energy, *F*_*m*_(*φ*, *θ*, *d*), by determining, computationally, the membrane shape satisfying the boundary conditions at the scaffold perimeters and minimizing the membrane bending energy. In the Supplemental Information we discuss in detail the steps of calculations illustrated by video V1 and the computational error estimation.

We determine the elastic energy of the scaffold interaction, *F*_*el*_(*d*), by finding for every given distance *d* the angle values, *φ* ^*^(*d*) and *θ* ^*^(*d*), which minimize the energy *F*_*m*_(*φ*, *θ*, *d*), and calculate the corresponding minimal energy value *F*_*min*_(*d*). Since we are interested in the energy of the scaffold interaction, the energy *F*_*el*_(*d*) is computed as *F*_*el*_(*d*) = *F*_*min*_(*d*)—*F*_*min*_(*∞*), where *F*_*min*_(*∞*)corresponds to large scaffold separations.

We next compute the free energy of the scaffold interaction, *F*(*d*). According to (Eq. 5), this requires computation of the bending energy, *F*_*m*_(*φ*, *θ*, *d*), for all possible values of the angles *φ* and *θ*. We vary the orientation angle *φ* in the whole range 0 < *φ <* 90°. For technical reasons, we change the tilt angle *θ* within a range limited by deviation of the energy *F*_*m*_ from its minimal value, *F*_*el*_(*d*), by not more than 4*k*_*B*_ *T*, which must give a sufficient accuracy of integration in (Eq. 5). The results are then interpolated to give the energy *F*_*m*_(*φ*, *θ*, *d*) over the entire range of *θ*. The resulting function is used for numerical integration according to (Eq. 5).

Finally, we determine the entropic contribution to the scaffold interaction, *F*_*ent*_(*d*), substituting the computed energies into (Eq. 6).

## Results

### Shallow protein scaffolds with anisotropic curvature

We start by analyzing scaffolds of circular shape, *r*_*a*_ = *r*_*b*_ = *a*, but different principal curvatures, *c*_*a*_ = 1/ *ρ*_*a*_, and *c*_*b*_ = 1/ *ρ*_*b*_ (Fig. 1B). We take the scaffold radius to be small compared to the principal curvature radii, *a*/ *ρ*_*a*_ ≪ 1, *a*/ | *ρ*_*b*_ |≪ 1 so that the contact angle *ϕ* is small, sin *ϕ* ≪ 1, which means that the scaffolds are shallow. For computations we take a specific ratio *a*/ *ρ*_*a*_ = 0.2, fix *c*_*a*_ and vary *c*_*b*_ within a range, *c*_*a*_ *≥ c*_*b*_ *≥ −c*_*a*_ which preserves the scaffold shallowness but represents different degrees of the scaffold curvature anisotropy. The limiting cases of *c*_*b*_ = *c*_*a*_ and *c*_*b*_ = −*c*_*a*_ correspond to spherically symmetric and saddle-like scaffolds, respectively, which have been analyzed analytically [19–21,26,30].

**Fig 2:**
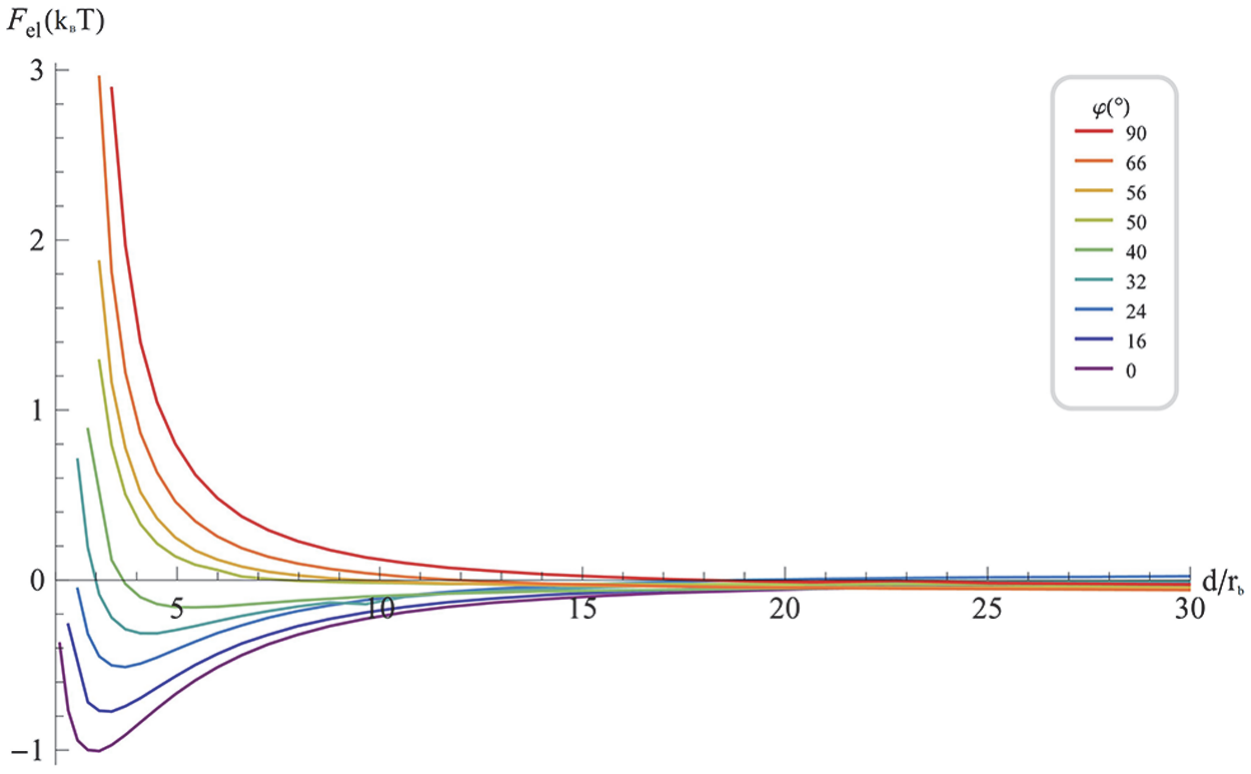
Elastic energy of shallow convex elliptical scaffolds with isotropic curvature (*ρ_a_ = ρ_b_* = *ρ*; *r_a_* = 1.5*r_b_* = 0.3*ρ*) as a function of the normalized distance between the scaffold centers, for various mutual orientation angle *φ*. The lowest energy for all distances corresponds to the orientation with *φ* = 0 where the scaffolds front each other by the faces with minimal contact angle. The energy is presented in units of *k*_*B*_ *T*, for *κ* = 20 *k*_*B*_ *T*.

Our computations show that for any given inter-scaffold distance, *d*, the inter-scaffold interaction energy depends on the mutual scaffold orientation. For convex scaffolds with *c*_*a*_ > *0* and *c*_*b*_ > *0* (or *c*_*a*_ > *0* and *c*_*b*_ > *0*), the minimal energy corresponds to such scaffold orientation where they front each other by their faces of the lowest contact angle *ϕ*, (Fig. 2). Saddle-like scaffolds characterized by one negative curvature, *c*_*a*_ > *0*, *c*_*b*_ < *0*, follow the same orientation rule in the major distance range, but exhibit a more complex mutual orientation at very small inter-scaffold distances.

**Fig 3:**
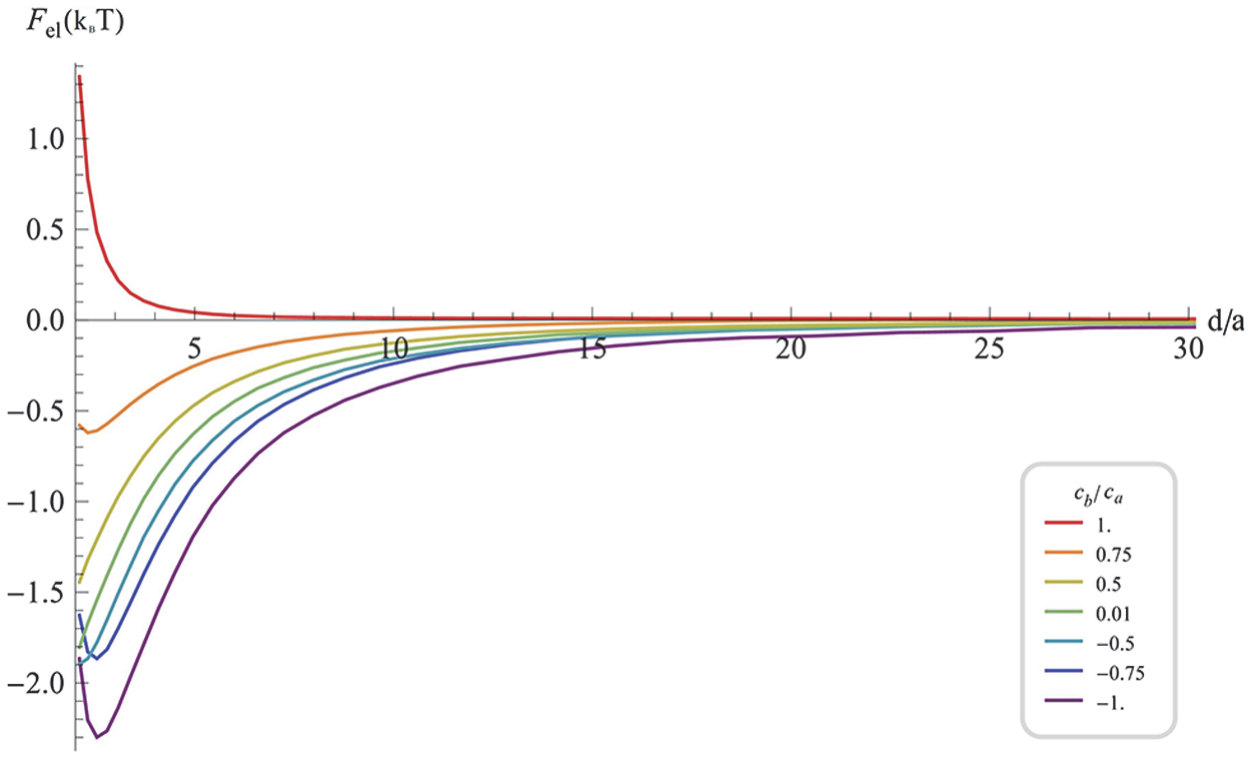
Elastic energy of interaction of shallow circular scaffolds as a function of the normalized distance between the scaffold centers for various degrees of anisotropy of the scaffold curvature characterized by *c_b_* ∕ *c_a_*. Scaffolds with isotropic curvature *c_b_* / *c_a_* = 1 exhibit a pure repulsion while any degree of anisotropy causes a contribution of attraction, which increases with the degree of anisotropy. The parameters are as in Fig. 2.

The elastic energy of the circular scaffold interaction, *F*_*el*_(*d*), corresponding to the optimal scaffold orientation, is presented in (Fig. 3) for various values of the second principal curvature *c*_*b*_. For the spherically symmetric scaffolds, *c*_*b*_ = *c*_*a*_ we obtain a monotonic repulsion over the whole distance range, which recovers the predictions of the analytical calculation for large distances (see Supplemental Information) [20,21,26]. Any anisotropy of the scaffold curvature results in attractive interaction at large distances, whereas at small distances the interaction remains repulsive. As a result, for anisotropic scaffolds, the energy profile as a function of *d* is non-monotonic with a minimum corresponding to an equilibrium value, *d* ^*^. This equilibrium distance *d* ^*^ rapidly decreases with the growing scaffold curvature anisotropy reaching values close to the particle size, *d* ^*^ = 2.36*a*, already for a relatively modest anisotropy of *c*_*b*_ = 0.75 *c*_*a*_. For smaller absolute values of *c*_*b*_, the energy minimum distance becomes smaller than the limiting accuracy of our computations. However, according to our estimations, the energy minimum disappears and the scaffold interaction is purely attractive up to zero distances for vanishing second curvature, *c*_*b*_ = 0, i.e. for scaffolds shaped as cylindrical elements, which are curved only along one principal axis. For negative values of *c*_*b*_ < 0, i.e. for scaffolds with saddle-like shapes, the repulsion at small distances and the related energy minimum at finites inter-scaffold distances reappear, as illustrated by the curves of (Fig. 2) corresponding to *c*_*b*_ = −0.75 *c*_*a*_ and *c* *_b_* = − *c*_*a*_.

**Fig 4:**
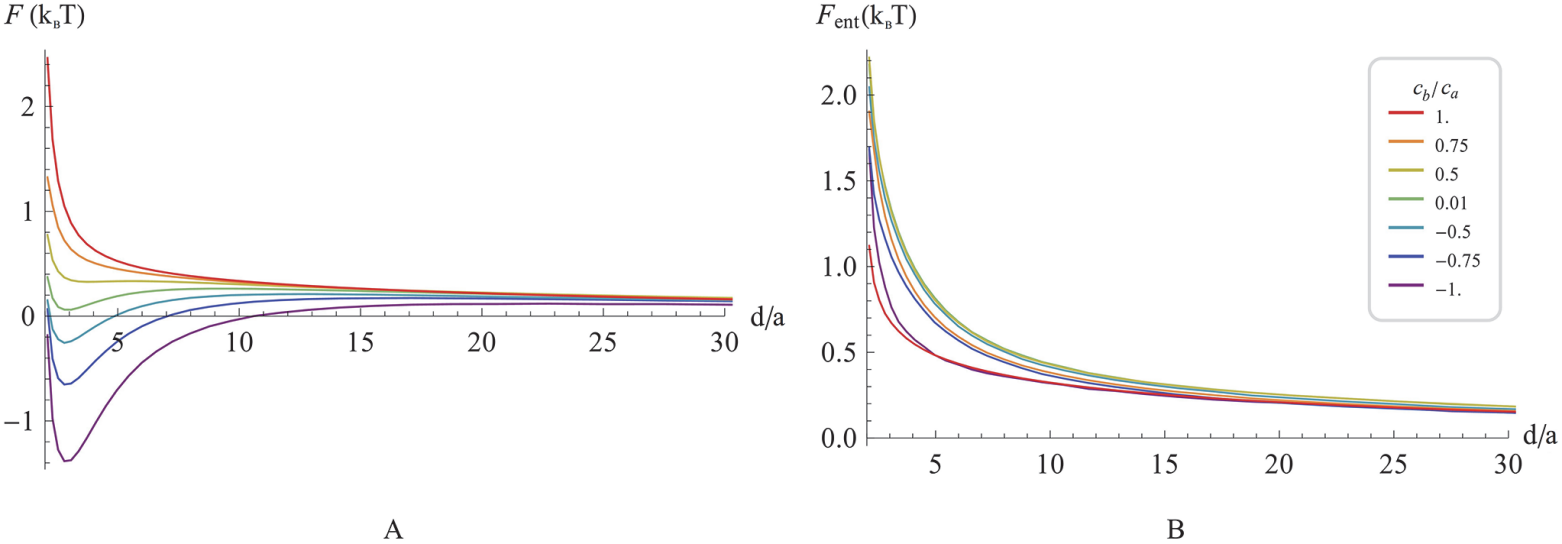
Free energy of interaction of shallow circular scaffolds (*r_a_* = *r_b_ = a*; *a* ∕ *ρa* ⪡ 1 ; *a* ∕ |*ρ_b_*| ⪡ 1) including the elastic and entropic contributions as a function of the normalized distance between scaffolds centers, for various degrees of the scaffold curvature anisotropy characterized by *c_a_* ∕ *c_b_*. (A). The total free energy. (B). Entropic part of the free energy according to (6). The parameters are as in Fig. 2.

The free energy of the scaffold interaction, *F*(*d*), for different degrees of the scaffold curvature anisotropy is presented in Fig. 4A. For all values of *c*_*b*_, the entropic effects included in *F*(*d*) turn the interaction at very short and at large distances to be repulsive (Fig. 4A). As a result, the overall inter-scaffold interaction is purely repulsive for low degrees of the scaffold anisotropy (Fig. 4A, *c*_*b*_ ≥ 0.75 *c*_*a*_) and characterized by a minimum corresponding to an equilibrium distance for stronger anisotropic scaffolds (Fig. 4A). The depth of the energy well increases with the scaffold anisotropy and reaches a values of ~1k_B_ T for saddle-like scaffolds (*c*_*a*_ = −*c*_*b*_) (Fig. 4A, purple curve).

The entropic contribution to the interaction energy, *F*_*ent*_(*d*), is presented in Fig. 4B. This interaction is purely repulsive and the energy has values of up to 2k_B_T. It is instructive to compare *F*_*ent*_(*d*) with the energy of the attractive interaction mediated by the membrane thermal undulations [20,21,31,32]. According to this comparison, the entropic effects related to the fluctuations of the scaffold orientations considered here can overcome those originating from the membrane undulations. Importantly, the presented computational results can only serve as a lower limit to the total entropic repulsion since for the purpose of simplicity we assumed the scaffold orientations to be mirror symmetrical. The full description of the free energy has to include also mirror asymmetric orientations, which, although energetically unfavorable, contribute to the entropic repulsion.

Next, we analyze the scaffold interaction for the cases where the scaffold shape anisotropy is related to an elongated elliptical shape of the scaffold projection on the initial membrane plane, *r*_*a*_ ≠ *r*_*b*_, rather than the difference in the scaffold principal curvatures. To this end, we compute the elastic energy of interaction, *F*_*el*_(*d*), between two scaffolds characterized by a fixed value of $r=\sqrt{r_{a}r_{b}}$, symmetric principle curvatures, *c*_*a*_ = *c*_*b*_ = 0.2∕ *r c*_*a*_, and different values of the aspect ratio *r*_*a*_ ∕ *r*_*b*_.

Concerning the scaffold mutual orientation, we obtain, similarly to the above case of anisotropic curvatures, that, for every distance *d*, the minimal energy corresponds to the scaffolds fronting each other by their faces of the lowest contact angle *ϕ*. This orientation corresponds to alignment of the scaffolds shortest axes. Thus, the mutual alignment of two convex scaffolds with the faces of the lowest contact angle fronting each other is a general prediction independent of whether the scaffold anisotropy is related to the deviation from the circular projection shape or inequality of the scaffold principle curvatures.

**Fig 5:**
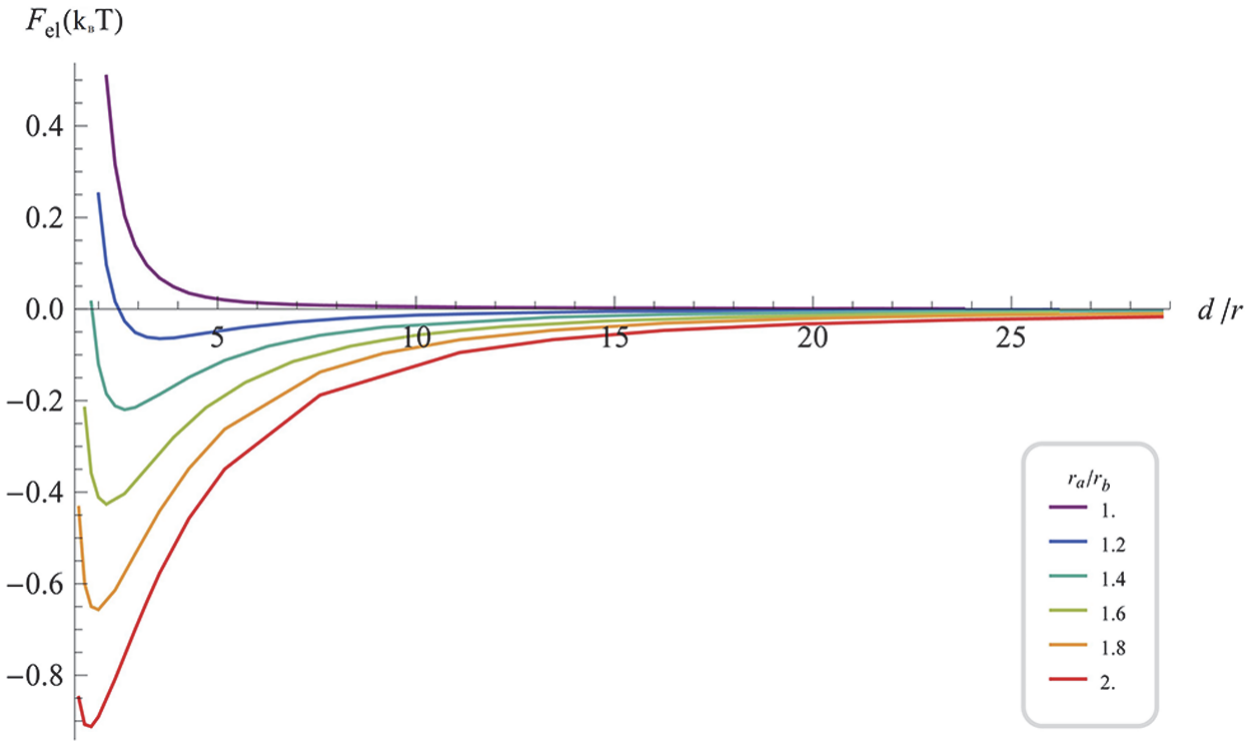
Elastic energy of shallow elliptical scaffolds with isotropic curvature (*ρ_a_* = *ρ_b_* = *ρ*; *r_a_* ∕ *ρ* ≪ 1; *r_b_* ∕ *ρ* ≪ 1) as a function of the normalized distance between scaffolds centers, for various values of the aspect ratio *r_a_/r_b_*. In all calculations$r=\sqrt{r_{a}r_{b}}=0.2\rho$. The curves are calculated for parallel orientation of the scaffold long axes, which corresponds to lowest energy at each distance. The parameters are as in Fig. 2.

The elastic interaction energy *F*_*el*_(*d*), corresponding to the optimal scaffold alignment and different degrees of the shape elongation is presented in Fig. 5. For symmetric scaffolds, *r*_*b*_ = *r*_*a*_, the interaction has been confirmed to be purely repulsive for all distances *d* (Fig. 5, Purple). The interaction between anisotropic scaffolds is attractive at large and repulsive and small inter-scaffold distances (Fig. 5). The equilibrium inter-scaffold distance corresponding to the energy minimum decreases and the depth of the energy well increases with growing anisotropy of the scaffold shape (Fig. 5).

### Highly curved proteins

The above computations for two shallow scaffolds revealed that the scaffold shape anisotropy of any kind results in an attractive component of the inter-scaffold interaction. This determines the existence of short equilibrium distances between and the corresponding optimal mutual orientation of the scaffolds. However, the depth of the energy well corresponding to the equilibrium distance has been predicted to be of the order of 1k_B_T, which is generally insufficient for overcoming the effects of the entropy of distribution all over the membrane and, hence, for holding the scaffolds close to each other.

To test the efficiency of the membrane mediated interactions between realistic scaffolds we performed computations for the scaffold parameters characterizing endophilin N-BAR domains. We took the dimensions of the scaffold projection on the membrane plane to be *r*_*a*_ = 6.5 *nm*, *r*_*b*_ = 1.5 *nm* and the curvature radius of the long axis to be *ρ*_*a*_ = 8.5 *nm* [7, 15, 41], and computed the elastic energy of the two scaffold interaction for various *ρ*_*b*_.

The computations show that, for the explored *ρ*_*b*_ range, such scaffolds mutually orient by aligning their short axes, which corresponds to fronting each other by their faces of lowest contact angle.

**Fig 6:**
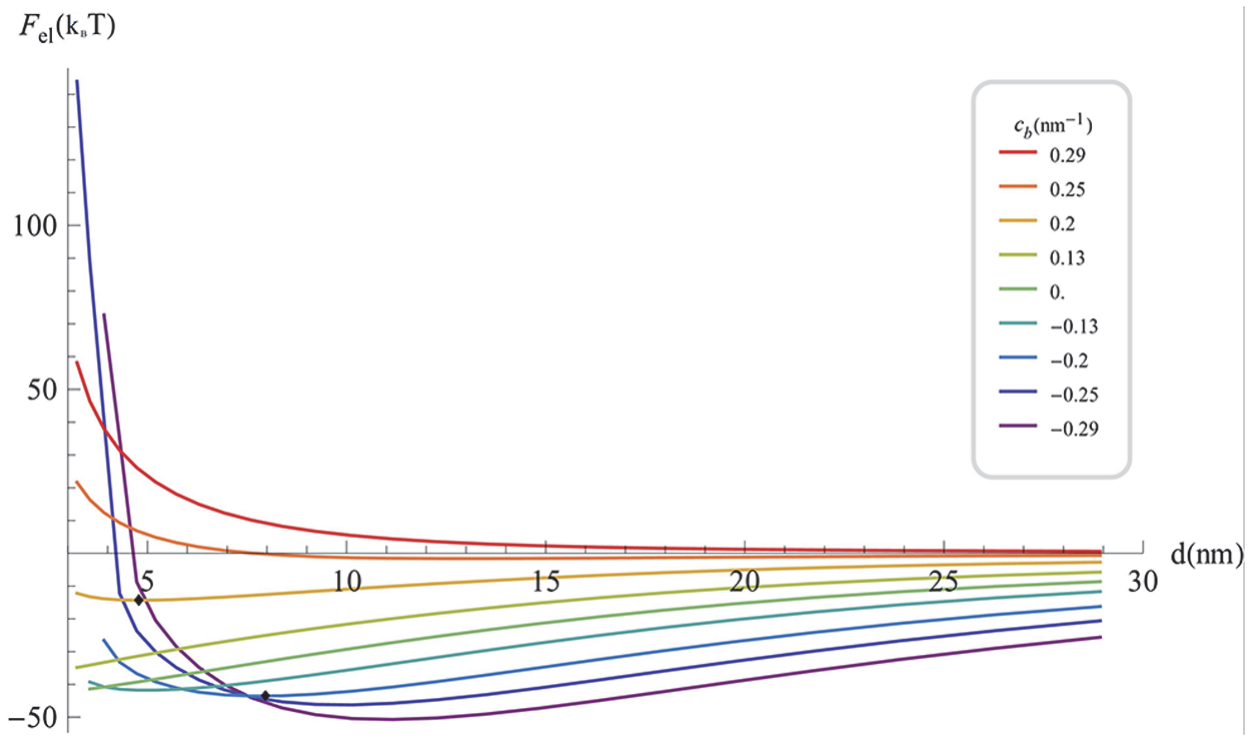
Elastic energy of highly curved scaffolds representing BAR domains (*r_a_* = 6.5 nm; *r_b_* = 1.5 nm; *ρ_a_* = 8.5 *nm* [5,31,32]) as a function of the distance between scaffolds centers, for various values of scaffold curvature along the short axis *c_b_*. The parameters are as in Fig. 2.

**Fig 7:**
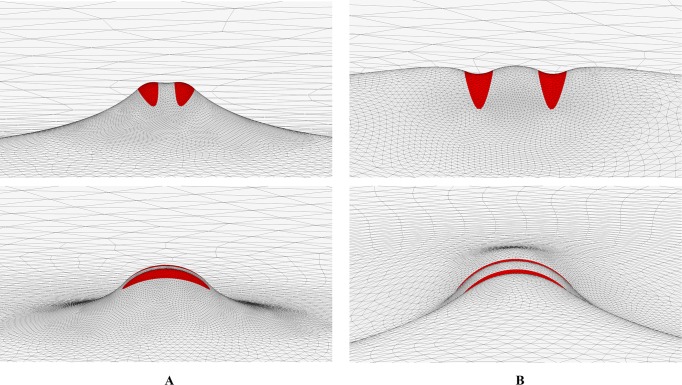
Calculated membrane shape of minimal energy in the vicinity of two scaffolds modeling BAR domains for two specific values of the scaffold short-axis curvature *c*_*b*_. (A). *c*_*b*_ = 0.2nm^-1^. (B) *c*_*b*_ = 0.2nm^-1^. Surface is shown in two orthogonal perspectives. Other parameters are as in Fig. 2.

Fig. 6 presents the elastic interaction energy at the optimal scaffold orientation as a function of the inter-scaffold distance, *F*_*el*_(*d*), for various values of *c*_*b*_ = 1 ∕ *ρ*_*b*_. For *c*_*b*_ < 0.2 nm^-1^, the elastic attraction between such highly elongated scaffolds is very strong compared to the above case of shallow scaffolds. As a result, the depth of the energy well corresponding to the equilibrium inter-scaffold distances, which are much smaller than the scaffold length, can reach few tens of k_B_T. The entropy contribution to the free energy is about 1k_B_T and can, therefore, be neglected in this case. Hence, the membrane-mediated interaction of realistically elongated and curved scaffolds is predicted to be sufficiently strong for guarantying an effective mutual ordering of the scaffolds and stably keeping them in a close proximity to each other. The computed membrane shapes in the vicinity of two such scaffolds characterized by specific values of *c*_*b*_ are presented in Fig.7.

## Discussion

We analyzed, computationally, the membrane mediated interactions between two strongly anisotropic and highly curved protein scaffolds in the whole range of inter-scaffold distances including, essentially, the distances much smaller than the scaffold dimension. The aim was to study the contribution of the membrane-mediated interactions to the self-assembly on the membrane surface of proteins known to drive shaping of intracellular membranes such as proteins of BAR-domain, dynamin and reticulon families. We modeled the protein scaffolds as elements of ellipsoidal (or hyperboloidal) surfaces with varying aspect ratio and anisotropic curvatures.

Our results address both the scaffold mutual orientation and their attractive-repulsive interactions. We obtained that two convex scaffolds tend to mutually orient such that they front each other by their faces of the lowest contact angle, *ϕ*, the latter defined as the angle between the normal vectors of the scaffold surface taken at the scaffold center and the scaffold circumference. Importantly, this result holds independently of the origin of the scaffold shape anisotropy, i.e. for both circular scaffolds with anisotropic curvature and isotropically curved scaffolds of elongated shapes.

Our computations showed that, while the membrane-mediated interaction between spherically symmetric scaffolds is repulsive for all inter-scaffold distances, any degree of the scaffold shape anisotropy of any kind results in an attractive component of this interaction provided that the scaffolds adopt the optimal mutual orientation. This is in agreement with previous analytical computations performed for large inter-scaffold distances and low degrees of anisotropy [22,27,28], and for purely saddle-like or cylindrical scaffolds [19,30,55]. For axi-symmetric scaffolds, as in [37], we were able to recover the specific character of the interaction dependence on the inter-scaffold distance predicted analytically [19,22,27,28,30], up to very small distances and for strongly curved scaffolds, i.e. for in the parameter ranges beyond the formal validity of the analytic solutions (see Supplemental Information). An important feature of our numerical results is that, already for modest degrees the scaffold shape anisotropy, they predict a relatively strong attractive interaction and the related equilibrium distances between the scaffolds much smaller than the scaffold dimension. Scaffolds characterized by zero contact angle along one direction are predicted to mutually attract up to zero distances.

Establishment of small or vanishing inter-scaffold distances is a prerequisite for the scaffold clusterization on the membrane surface, whose feasibility depends however on the depth of the energy well representing the scaffold-scaffold binding energy. Our results show that, while for shallow scaffolds this membrane-mediated binding energy is of the order of 1k_B_T, the strongly curved and anisotropic scaffolds imitating geometrically such real protein modules as BAR-domain can bind each other with energies of few tens of k_B_T, which guarantees a high efficiency of clusterization. Hence, the membrane-mediated interaction must be sufficient to drive self-assembly of the specialized proteins on the membrane surface into clusters and to determine a specific mutual orientation of these proteins within the clusters. Feasibility of linear aggregation of curvature-generating proteins such as N-BAR domains, which bend membranes by both scaffolding and hydrophobic insertions mechanisms, has been recently demonstrated by coarse-grained simulations [56].

The membrane-mediated interaction between scaffolds leading to their stable self-assembly on the membrane surface into ordered arrays may be of a major importance for shaping strongly curved intracellular compartments such as endoplasmic reticulum (ER), Golgi Complex and mitochondria (see for review e.g. [8]). For example, it has been proposed and confirmed experimentally that the ER tubules and sheets are sculptured by linear arrays of aligned arc-like scaffolds formed by oligomers of reticulons and DP1/Yop1 family proteins [57–59]. According to the results of the present work, formation of such arrays may be driven by the scaffold attractive and orientational interactions mediated by the membrane bending deformations. It has to be emphasized, however, that the membrane-mediated interaction between multiple membrane inclusions is not pairwise [60]. Therefore, while the present results can serve as a qualitative assessment of the scaffold self-assembly pathways, a quantitative analysis of these processes requires further modeling and computations.

### Entropic repulsion between scaffolds

In our analysis, we addressed a contribution to the two-scaffold interaction related to the entropy of fluctuations of the scaffold orientation with respect to each other and of the scaffold tilting with respect to the initial membrane plane. This interaction, which remained unaddressed by the previous works, has a repulsive character. The physical origin of this interaction is in a mutual limitation by the approaching scaffolds of the number of available orientations and the related reduction of the system entropy. Here, we compute the entropy effects of only the symmetric mutual orientations of the scaffolds, meaning that the results underestimate the strength of the related interaction.

This entropic repulsion counteracts the well-explored attractive interaction, which originates from the entropy of membrane undulations [20,21,31,33]. According to our results, the orientational entropic repulsion considered here can overcome the entropic attraction related to the membrane undulations.

### Model assumptions

We performed the computations for two scaffolds embedded into a closed membrane whose size, R, exceeds by four orders of magnitude the scaffold linear dimensions, *r*_*a*_ and *r*_*b*_, and the inter-scaffold distance, *d*. In spite of the scale difference, a question remains whether the obtained results are equivalent to the scaffold interaction within an initially absolutely flat membrane contacting a lipid reservoir with vanishing lateral tension. Indeed, in the absence of the lateral tension, the membrane bending deformations are long range and decay with the distance according to a power low [26]. Hence, basically, the shape of the whole membrane must undergo some extent of deformation [61] upon the scaffold embedding, which may result in a dependence of the scaffold interaction on the boundary conditions imposed on the membrane shape at large distances. The effects of membrane closure on the inclusion interaction for relatively small membrane dimensions were analyzed in [62].

To check this issue in our system, we computed the elastic energy of the inter-scaffold interaction in the presence of low lateral tension, *γ*, which results in an exponential decay of the membrane deformations [26] and, hence, must eliminate the dependence of the results on the membrane boundary conditions at large distances. The scaffold parameters were taken to be *r*_*a*_ = 0.23 *ρ*_*a*_; *r*_*b*_ = 0.175*ρ*_*a*_; *ρ*_*b*_ = *ρ*_*a*_ and the tension value was chosen such that the characteristic length of the exponential decay of deformation, $\;\xi =\sqrt{\kappa /\gamma }$ [26], was much smaller than the membrane dimension *ξ* ≈ 10^–2^ R, but exceeds by two orders of magnitude the scaffold dimensions *r*_*a*_ and *r*_*b*_, and the inter-scaffold distance *d*. We found that while the tension-mediated interaction between the scaffolds is always repulsive and depends on the strength of the membrane tension *γ*, the curvature contribution to the interaction remains equal to that we obtained at zero tension. Hence, closure of large membrane does not influence the curvature-mediated interaction between two scaffolds.

According to another assumption, we considered only mirror symmetric mutual orientations of the scaffolds. This was verified by computation of the interaction energy for circular saddle-like scaffolds with *ρ*_*b*_ = −*ρ*_*a*_ adopting asymmetric orientations *φ*_*1*_ ≠ *φ*_*2*_, *θ*_*1*_ *≠ θ*_*2*_. We found the energies larger than for the symmetric orientations.

## Supporting Information

S1 Text Membrane-mediated interaction between strongly anisotropic protein scaffolds: Supplemental Information. (DOCX)

S1 Video Time-lapse representation of the calculation process for fixed scaffold shape. The calculation procedure involves finding the shape of minimal bending energy of the membrane for various orientations of and distances between the scaffolds. Each frame in the video represents the shape of minimal energy for a different configuration. The configurations follow in the same order as in the calculation. The “spreading” of vertices along the membrane stems from repeating averaging of the facets areas, as used in every calculation.
(MP4)

S1 Fig Evolution of calculation of the minimal shape, from the initial setup (Left) to the final minimal shape (Right). Insets show close-up views on the scaffolds.
(TIF)

S2 Fig Computational error as a function of the maximal dihedral angle. (TIF)

S3 Fig Log-log plot of the elastic energy of interaction between axi-symmetric scaffolds (*ρ*_*a*_ = *ρ*_*b*_ *≡ ρ; r*_*a*_ = *r*_*b*_ ≡ *r*) as a function of the inter-scaffold distance for various scaffold sizes *r* ∕ *ρ*. Circles represent the calculated data while the straight lines are calculated from Eq. S1 for *k* = 20*k*_*B*_ *T*. The black horizontal line shows the magnitude of computational error, described in the previous section, which means that the computational values below this line are unreliable.
(TIF)

S4 Fig Log-log plot of the elastic energy of interaction between shallow scaffolds (dots), as shown in the main text (Figs. 3 and 5), fitted to Eq. S2 (solid lines). Left panel: results for scaffolds with circular projection but different principle curvatures. Right panel: results for scaffolds of equal principle curvatures but elliptical projections.
(TIF)
