# Supplementary material for: Membrane-Mediated Interaction between Strongly Anisotropic Protein Scaffolds
Source: PLoS Comput Biol. 2015 Feb 24;11(2):e1004054. doi: 10.1371/journal.pcbi.1004054 (PMC4339200; doi:10.1371/journal.pcbi.1004054)
Supplement: S1 Text — (DOCX) [file pcbi.1004054.s001.docx]

Membrane-mediated interaction between strongly anisotropic protein scaffolds

**Supplemental Information**

All the calculations in this work were done using K. Brakke’s Surface Evolver (SE) [54]. SE is a numerical tool that uses a triangulated network to model a 2-dimensional surface in a 3-dimensional space and minimizes its energy according to a specified Hamiltonian. We use the Helfrich Hamiltonian (Eq. 3) in order to minimize the curvature energy of a closed membrane with two protein scaffolds embedded into it, as described in the main text. Here we show the calculation steps, alongside an estimation of the computational errors.

**Calculation steps**

We first find the shape of the membrane that minimizes the Helfrich energy under the constraints of fixed scaffolds in a certain configuration, which is defined by the distance between the scaffolds, $d$, and the two orientation angles, $\theta$ and (see main text). We start by fixing four vertices along the perimeter of each scaffold, and define eight more vertices on the closed membrane that define the initial shape (Fig. S1). Then, by consecutive steps of the SE commands ‘refine’ (add a vertex at the center of each facet edge), ‘g $n$’ (make $n$ iterations of gradient descent) and ‘hessian_seek’ (minimize the energy using the Hessian matrix) we let the membrane converge to a shape of minimal energy (Fig. S1), referred to as the minimal shape.

Since the accuracy of calculations depends on the ratio between the size of the triangles constituting the surface and the radius of the local curvature, and because the bending deformations close to the scaffolds are generally larger than for the rest of the surface of the final shape, we further refine the triangulation at radii $2\rho_{a}$ and $10\rho_{a}$ around each scaffold. This way we ensure a sufficiently fine triangulation to match our accuracy criteria (see discussion on accuracy below). The convergence condition of the computations is fulfilled when a single hessian seek step reduces the total energy by less than $10^{-7}\kappa$ and all the accuracy criteria are satisfied (see below).

As the initial configuration between the scaffolds we took a fixed orientation angle and a zero tilt angle $\theta=0$. Our goal was to analyze the scaffold interaction in a broad range of the inter-scaffold distances, $d$, up to complete touch between the scaffold boundaries. At the same time, as the inter-scaffold distance decreases, the effective curvatures, generated by the computational procedure in the course of the membrane shape variations aimed at the energy minimization, increases. This leads to problems with convergence of the computations. The reason for that is a mismatch between the minimal possible scale of triangulation of the membrane surface and the thickness of the membrane strip left between the scaffolds. To avoid this difficulty we limited the analysis by the situations where the distance between the closest points of the scaffolds boundaries is not smaller than $0.1r_{b}$, so that the residual membrane strip is sufficiently large for enabling a refinement of the triangulation necessary for the computation convergence. The corresponding minimal distance between the centers of the circular scaffolds is $d=2.1r_{b}$, which includes the radii of the two scaffolds in addition to the thickness of the inter-scaffold membrane gap. This limitation for the minimal distance between the scaffolds enables using a discrete approach for computational treatment of a continuous membrane surface.

After we find the minimal shape for the initial configuration, we vary the tilt angle in $\Delta\theta=1^{\circ}$ steps by using a simple rotational transformation for all vertices belonging to the scaffold surfaces. For each values of $\theta$ we find the minimal shape and its energy. We change the tilt angle, $\theta$, until the energy reaches its minimum, , at an optimal value . We then continue variation of $\theta$ beyond $\theta_{opt}$ until the energy exceeds by $4k_{B}T$. In the next step, while keeping fixed, we increase the distance, $d$, between the scaffolds by $\delta d$ constituting 5% of $d$. We then repeat the computations for different tilt angles, $\theta$, according to the scheme above for the new distance $d+\delta d$. In this way we find the elastic energy, $F_{m}\left(\theta,d\right)$, as a function of the angle $\theta$ and the distance, $d$, for a given orientation angles . Video V1 shows a time-lapse representation of this calculation. Repeating the whole computation for different values of the orientation angle, , we obtain the energy landscape $F_{m}(\theta,d,$ ).

Minimization of the energy $F_{m}(\theta,d,$ ), with respect to the angles $\theta$ and for every distance $d$ yields the elastic energy of the repulsive-attractive interaction, $F_{el}\left(d\right)$. The obtained energy landscape enables determination of the system free energy, $F\left(d\right)$, by its substitution into (Eq. 5).

**Accuracy criteria and error estimation**

In order to have a reliable measure of the accuracy of representation in this computation of a continuous surface by a triangulated one, we first quantify the error in calculating the minimum energy and its dependence on the parameters of the model. We found that the most crucial accuracy indicator is the maximal edge dihedral angle [S1], defined as a maximal angle between the normal vectors of any two adjacent facets of the triangulated surface. To find the dependence of the error on the maximal dihedral angle we consider two shallow scaffolds with isotropic curvature $\rho_{a}=\rho_{b}$ and elliptical projection, $r_{a}=1.5r_{b}=0.3\rho_{a}$, and find, according to the procedure described in the previous section, the energy of the minimal shape for fixed angles, $\theta=0$ and , and a fixed inter-scaffold distance $d$. Since the calculated energy depends on the exact triangulation used, we repeat the calculation for 84 randomly oriented initial shapes of the surface, which leads to 84 different triangulations of the surface (although the overall minimal shape was not changed noticeably) and 84 different calculated values for the energy of the minimal shape. We define the computational error as the standard deviation of the calculated energy from the average.

In Fig. S2 we show the standard deviation of the calculated energies as a function of the average maximal dihedral angle. We tested this relationship for four different distances between scaffolds, where the maximal curvature, and, hence, also the maximal dihedral angle, increases as the distance between the scaffolds increases. According to Fig. S2, we set the upper limit of the error in the calculated energy to be 0.04$k_{B}T$, by limiting our calculations for a maximal dihedral angle not larger than 4°.

Another accuracy indicator is the fineness $f=A/l^{2}$ of the surface triangulation [S1], where $A$ is the total area of the surface and $l$ the longest facet edge length. We set the required fineness$f$ by performing the energy computation for an ideal sphere and requiring that the resulting numerical value of the energy deviates from the exact analytical values of $8\pi\kappa$ by not more than 0.02%. This requirement corresponding to guarantees the energy computational error to be below 0.04$k_{B}T$.

In addition to the above requirements [S1] we found that, since the discrete curvature is calculated in SE at the vertices and not on the edges, a vertex that connects very short facet edges with relatively long ones leads to instability and large errors in the calculation. To avoid this problem, we define the edge ratio of a vertex as the ratio between the longest and shortest edges connected to the same vertex, and require the maximal edge ratio to be not larger than 7.

In all calculation steps described in the previous section we omit the results that do not satisfy all three the accuracy criteria above, i.e. the fineness, the maximum dihedral angle and the maximum edge ratio.

**Comparison with analytic results**

To verify the computational procedure we used, we compare our numerical results with the previously obtained analytical results. First, we test the curvature-mediated repulsion between axially symmetric scaffolds ($r_{a}=r_{b}\equiv r ; \rho_{a}=\rho_{b}\equiv\rho$). This interaction was found, for large distances $d\gg r$ and shallow scaffolds $\rho\gg r$, to follow

| $F_{el}=8\pi\kappa\alpha^{2}\left(\frac{r}{d}\right)^{4}$ | (S1) |
| --- | --- |

where $\alpha=\arcsin\left(r/\rho\right)$ is the contact angle along the scaffold boundary [20,22,26,39,55]. Fig. S3 shows the elastic energy, calculated by our procedure, as a function of the normalized distance $d/r$, for axi-symmetric scaffolds with various values of $r/\rho$. As discussed in the main text, the interaction is purely repulsive and, as shown in Fig.S3, the numerical results (circles) are in a good agreement with the analytical relationship (Eq. S1) (solid lines). As noted in [37], this agreement extends to the cases of small inter-scaffold distances and strongly curved scaffolds, which are beyond the validity of the analytical derivation [20,22,26,39,55].

We further tested the computational results obtained for shallow anisotropic scaffolds. As discussed in the main text, any kind of anisotropy in the scaffolds shape is predicted to add an attractive component to the elastic interaction, which decays at large distances, $d\gg r$, as $-\left(r/d\right)^{2}$ [6,7,9]. Fig. S4 presents fitting of the computational results for shallow non-isotropic scaffolds (as presented in Figs. 3,5 of the main text) at large distances to the analytical expression

| $F_{el}=-A\left(r/d\right)^{2},$ | (S2) |
| --- | --- |

where $A$ is a real positive fitting parameter. For both types of the scaffold anisotropy we addressed here, i.e. for scaffolds with elliptical rather than circular projection, $r_{a}\neq r_{b}$, and for scaffolds with unequal radii of principal curvatures, $\rho_{a}\neq\rho_{b}$, our computational results recover for large distances $d\gg r$ the analytically predicted scaling (Eq. S2) [27,39,55]. At closer distances additional repulsive terms must be taken into account.

**Supplemental references**

S1. Hsu L, Kusner R, Sullivan J (1992) Minimizing the Squared Mean Curvature Integral for Surfaces in Space Forms. Experimental Mathematics 1: 191-207.

**Supplemental figure legends**

Fig S1. Evolution of calculation of the minimal shape, from the initial setup (Left) to the final minimal shape (Right). Insets show close-up views on the scaffolds.

Fig. S2. Computational error as a function of the maximal dihedral angle.

Fig. S3. Log-log plot of the elastic energy of interaction between axi-symmetric scaffolds ($\rho_{a}=\rho_{b}\equiv\rho$ ; $r_{a}=r_{b}\equiv r$) as a function of the inter-scaffold distance for various scaffold sizes $r/\rho$. Circles represent the calculated data while the straight lines are calculated from Eq. S1 for $\kappa=20k_{B}T$. The black horizontal line shows the magnitude of computational error, described in the previous section, which means that the computational values below this line are unreliable.

Fig. S4. Log-log plot of the elastic energy of interaction between shallow scaffolds (dots), as shown in the main text (Fig. 3 and 5), fitted to Eq. S2 (solid lines). Left panel: results for scaffolds with circular projection but different principle curvatures. Right panel: results for scaffolds of equal principle curvatures but elliptical projections.
