# Supplementary figures and images for: Membrane-Mediated Interaction between Strongly Anisotropic Protein Scaffolds
Source: PLoS Comput Biol. 2015 Feb 24;11(2):e1004054. doi: 10.1371/journal.pcbi.1004054 (PMC4339200; doi:10.1371/journal.pcbi.1004054)

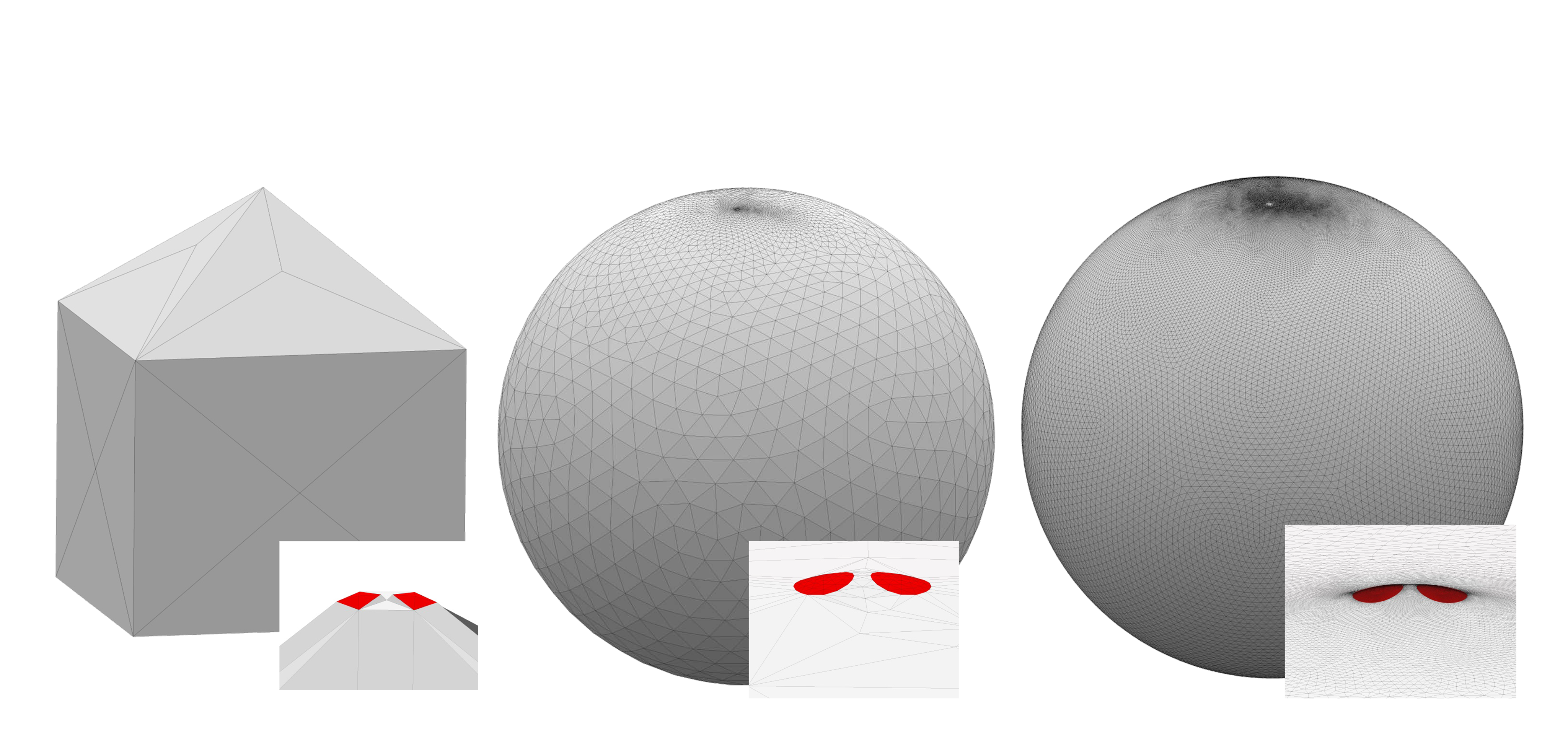

Supplement: S1 Fig — Insets show close-up views on the scaffolds. (TIF) [file pcbi.1004054.s003.tif]

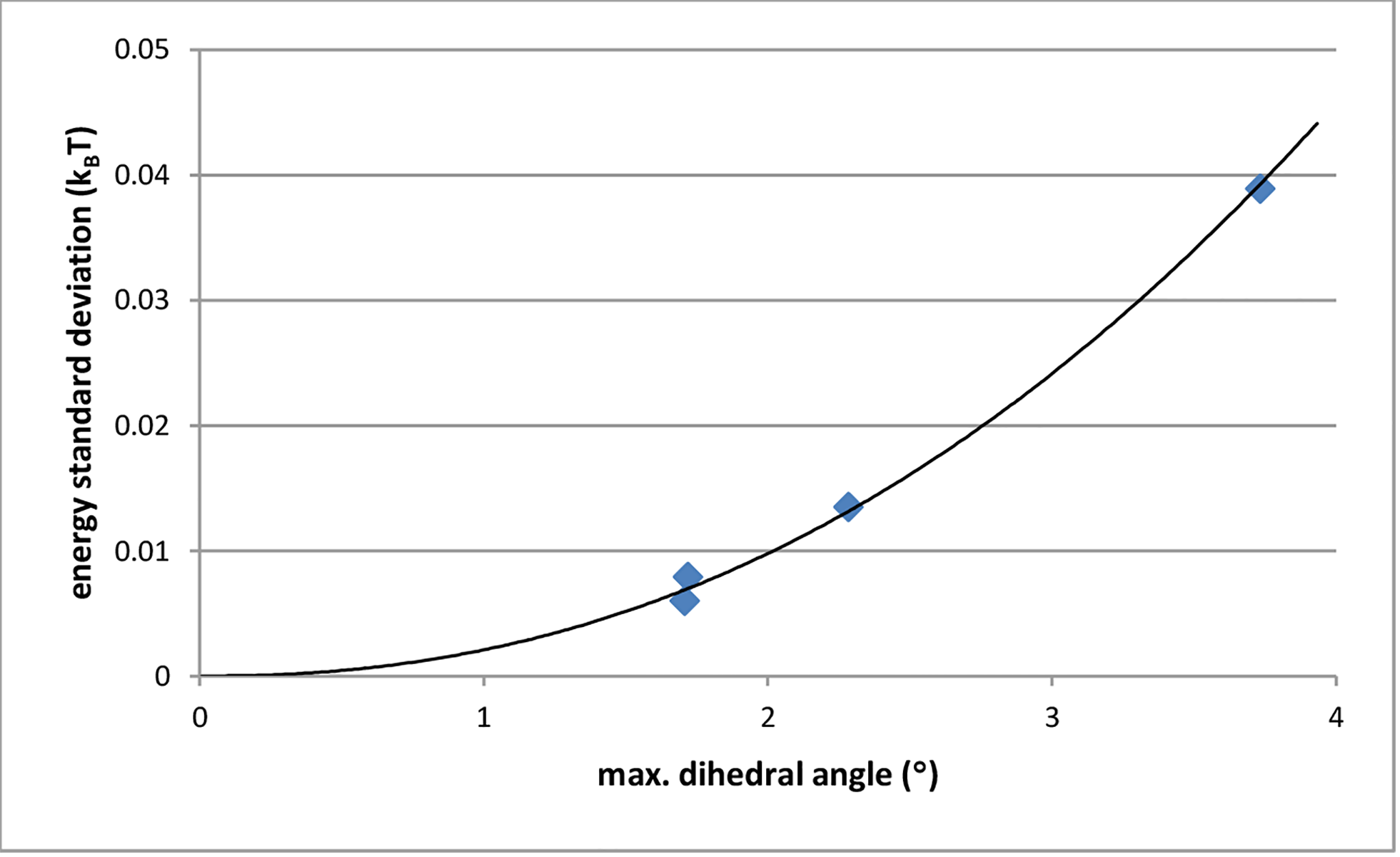

Supplement: S2 Fig — (TIF) [file pcbi.1004054.s004.tif]

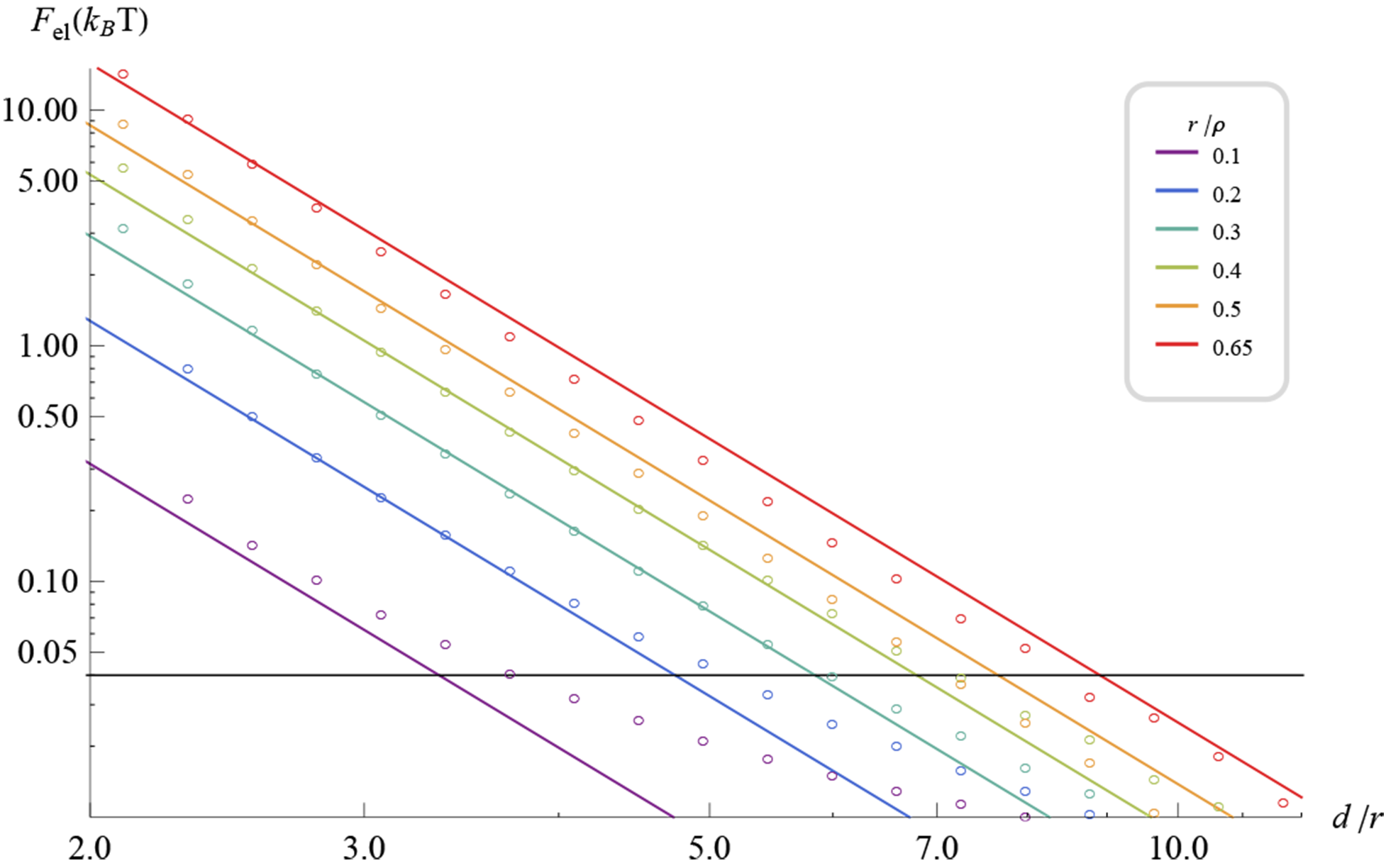

Supplement: S3 Fig — Circles represent the calculated data while the straight lines are calculated from Eq. S1 for k = 20k B T. The black horizontal line shows the magnitude of computational error, described in the previous section, which means that the computational values below this line are unreliable. (TIF) [file pcbi.1004054.s005.tif]

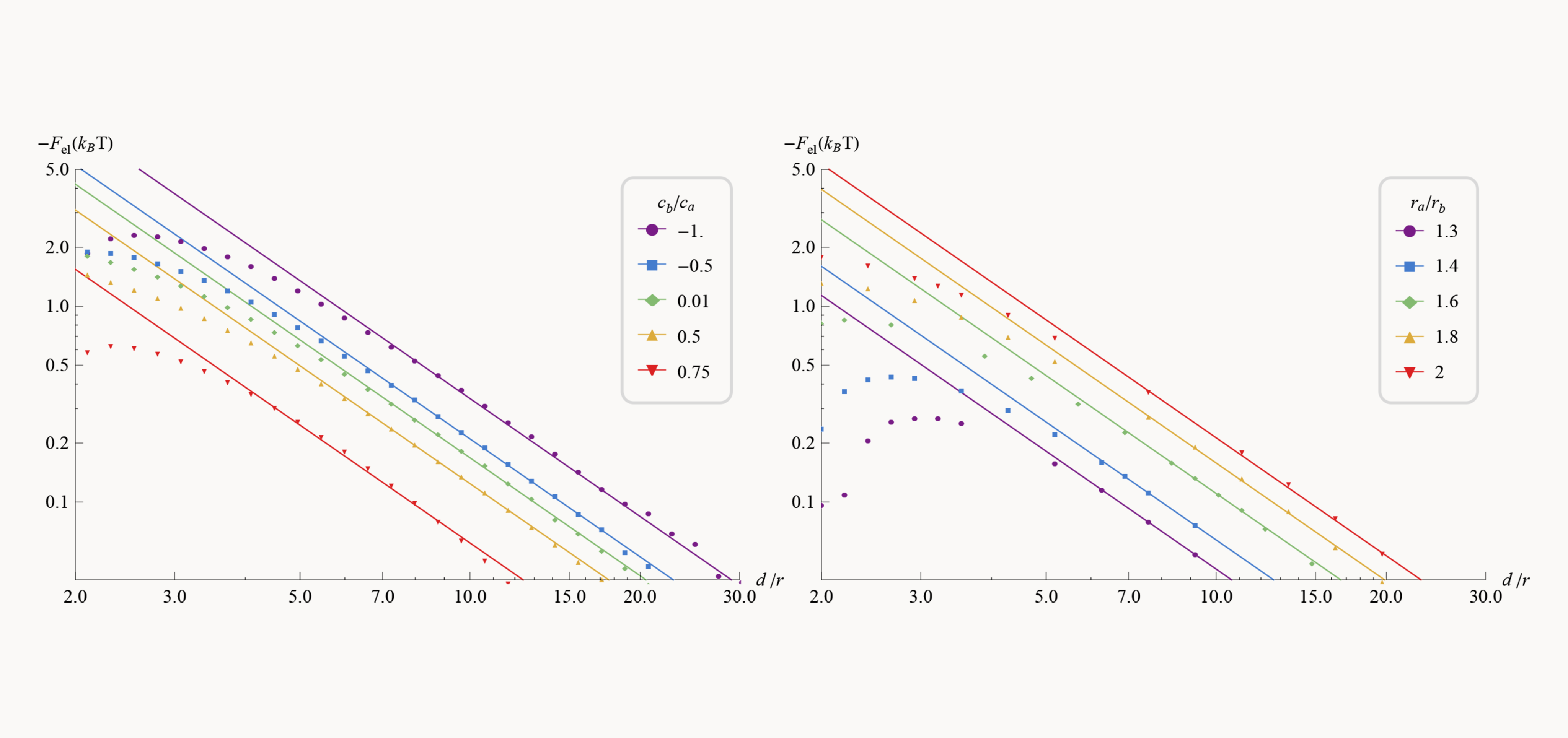

Supplement: S4 Fig — Left panel: results for scaffolds with circular projection but different principle curvatures. Right panel: results for scaffolds of equal principle curvatures but elliptical projections. (TIF) [file pcbi.1004054.s006.tif]
